# Systems biology analysis reveals NFAT5 as a novel biomarker and master regulator of inflammatory breast cancer

**DOI:** 10.1186/s12967-015-0492-2

**Published:** 2015-05-01

**Authors:** Andrea Remo, Ines Simeone, Massimo Pancione, Pietro Parcesepe, Pascal Finetti, Luigi Cerulo, Halima Bensmail, Daniel Birnbaum, Steven J Van Laere, Vittorio Colantuoni, Franco Bonetti, François Bertucci, Erminia Manfrin, Michele Ceccarelli

**Affiliations:** Department of Pathology, Mater Salutis Hospital, Legnago, Italy; Department of Science and Technology, University of Sannio, Benevento, Italy; Qatar Computing Research Institute (QCRI), Qatar Foundation, Doha, Qatar; Department of Pathology and Diagnosis, University of Verona, Verona, Italy; Department of Molecular Oncology, Institut Paoli-Calmettes, U1068 Inserm, Marseille, France; Bioinformatics Laboratory, BIOGEM, Ariano Irpino, Avellino, Italy; Department Medical Oncology, University of Antwerp, Antwerpen, Belgium

**Keywords:** Inflammatory breast cancer, Gene regulatory network, Systems biology, NFAT5, MGA, CTNNB1

## Abstract

**Background:**

Inflammatory breast cancer (IBC) is the most rare and aggressive variant of breast cancer (BC); however, only a limited number of specific gene signatures with low generalization abilities are available and few reliable biomarkers are helpful to improve IBC classification into a molecularly distinct phenotype. We applied a network-based strategy to gain insight into master regulators (MRs) linked to IBC pathogenesis.

**Methods:**

*In-silico* modeling and Algorithm for the Reconstruction of Accurate Cellular Networks (ARACNe) on IBC/non-IBC (nIBC) gene expression data (*n* = 197) was employed to identify novel master regulators connected to the IBC phenotype. Pathway enrichment analysis was used to characterize predicted targets of candidate genes. The expression pattern of the most significant MRs was then evaluated by immunohistochemistry (IHC) in two independent cohorts of IBCs (*n* = 39) and nIBCs (*n* = 82) and normal breast tissues (*n* = 15) spotted on tissue microarrays. The staining pattern of non-neoplastic mammary epithelial cells was used as a normal control.

**Results:**

Using *in-silico* modeling of network-based strategy, we identified three top enriched MRs (NFAT5, CTNNB1 or β-catenin, and MGA) strongly linked to the IBC phenotype. By IHC assays, we found that IBC patients displayed a higher number of NFAT5-positive cases than nIBC (69.2% vs. 19.5%; *p*-value = 2.79 10^-7^). Accordingly, the majority of NFAT5-positive IBC samples revealed an aberrant nuclear expression in comparison with nIBC samples (70% vs. 12.5%; *p*-value *=* 0.000797). NFAT5 nuclear accumulation occurs regardless of WNT/β-catenin activated signaling in a substantial portion of IBCs, suggesting that NFAT5 pathway activation may have a relevant role in IBC pathogenesis. Accordingly, cytoplasmic NFAT5 and membranous β-catenin expression were preferentially linked to nIBC, accounting for the better prognosis of this phenotype.

**Conclusions:**

We provide evidence that NFAT-signaling pathway activation could help to identify aggressive forms of BC and potentially be a guide to assignment of phenotype-specific therapeutic agents. The NFAT5 transcription factor might be developed into routine clinical practice as a putative biomarker of IBC phenotype.

**Electronic supplementary material:**

The online version of this article (doi:10.1186/s12967-015-0492-2) contains supplementary material, which is available to authorized users.

## Background

Inflammatory breast cancer (IBC) is a rare disease that accounts approximately for 5% of breast cancers [1]. Because of its biological and clinical features of rapid progression including high invasiveness, neoangiogenesis, and frequent local and metastatic recurrences, IBC is the most aggressive form of primary breast cancer. Despite progresses in the multidisciplinary treatment, the prognosis is poorer than that of non-inflammatory breast cancer (nIBC), with a 5-year overall survival rate of only 40%, compared with 85% in stage III nIBC patients. IBC diagnosis is based on a combination of clinical information, typically combining fast development (<6 months) in the affected breast of specific signs and symptoms, such as pain, erythema, edema, reddening, “peau d’orange” of the overlying skin, abnormalities of the nipple and udder enlargement and induration [2].

The low prevalence of IBC, and the small size of diagnostic biopsy specimens have been obstacles in understanding of IBC pathogenesis. To date, current treatments are based on multimodal approaches, are non-specific for IBC, and do not result in long-term eradication of the disease. Previous studies have identified several genes and pathways that might contribute to the IBC phenotype. Approximately 57% of IBCs are estrogen receptor (ER) and progesterone receptor (PR) negative, whereas about 30% are triple-negative breast cancers (TNBC) [3]. IBCs present often mutations in the *TP53* tumor suppressor, overexpression of CDH1 and angiogenic factors such as VEGF, FGF2 and VEGFR1 [4]. Using high-throughput molecular analyses, Van Golen et al. [5] reported frequent overexpression of RHOC GTPase and loss of expression of WISP3/LIBC (Lost in Inflammatory Breast Cancer). IBCs have also a higher Ki-67 expression of than nIBCs [6]. Using an integrated analysis of gene expression and array-based comparative genomic hybridization (aCGH), 24 potential IBC-specific oncogenes have been identified, which could be involved in IBC aggressiveness [7]. More recently, the World IBC Consortium was founded to foster collaborations between research groups focusing on IBC with the aims of establishing the molecular profile of IBC using a wide number of samples and of searching for gene signatures associated with survival and response to neo-adjuvant chemotherapy [8]. The analysis of about four hundreds of whole-genome mRNA expression profiles revealed that IBC is transcriptionally heterogeneous, that all molecular subtypes described in nIBC are also detectable in IBC, albeit with a different frequency, and identified down-regulation of TGFβ as biologically relevant [9]. However, these advances have not yet led to clinical applications, and the need to identify clinical IBC biomarkers to improve diagnosis and treatment persists.

Gene expression changes and the coordination of cellular behavior depend on the activity of transcription factors (TFs) acting as master regulators (MR). In a number of cancer types, using context-specific network strategies has revealed the role of MRs. For example, the role of STAT3 and CEBP/B as responsible for the mesenchymal transformation in glioblastoma was evidenced by *de novo* reconstruction of the transcriptional network underlying the observed phenotype [10]. Master regulators of FGFR2 signaling were recently identified in a similar fashion by using several datasets for discovery and validation [11]. Gene networks and MRs have also been studied in stem cells [12,13]. Such identification of MRs is called Master Regulator Analysis and is based on the enrichment of the TF regulon (the set of predicted TF targets) with respect to a specific gene signature of the considered phenotype [10,14]. The basic information needed to apply this network-based strategy relies on the availability of a context-specific TF-centric regulatory network that can be computed via inferential statistics approaches using many gene expression data. *De novo* gene network inference can be both unsupervised [15,16] and supervised [17] and can be based on a number of heuristics [18,19].

Here, we describe a network-based strategy to identify TFs acting as MRs in IBC. We show that the nuclear expression of the Nuclear Factor of Activated T-Cell 5 (NFAT5) TF is a peculiar feature of IBC, which could be used as a potential biomarker of this disease and a possible candidate for treatments.

## Methods

### Microarray gene expression dataset and supervised analysis

To better understand the gene expression signature and identify potential transcriptional regulators of IBC aggressiveness, we used, as learning set, a previously published gene expression dataset [7] including 197 breast cancer samples from IPC Marseille-France (63 IBCs and 134 nIBCs) and available from the NCBI’s Gene Expression Omnibus (GEO) portal (GSE23720) and the validation set [9] including 96 samples from the General Hospital Sint-Augustinus (Antwerp, Belgium; 41 IBCs and 55 nIBCs). The Affymetrix CEL files of both datasets were converted to normalized expression value using Robust Multi-Array Average (RMA) method provided by “affy” Bioconductor package.

For the supervised analysis of gene expression profiles between the IBC and nIBC groups of the learning set, differentially expressed (DE) probe-sets were first filtered for absolute fold change ≥ 1.5. Statistical analysis was then applied to these filtered probe-sets using the Student’s *t*-test, with *p* ≤ 0.05. The robustness of the resulting gene-list was tested in the Belgium independent validation set. A classifier of samples based on the expression of the differentially expressed probe sets was built by measuring the Pearson correlation (*r* thereafter designated as IBC score) of each sample with the IBC centroid, defined as the average corresponding expression profile of all IBCs from the learning set: samples with *r* > 0 were classified as “IBC-like” and those with *r* < 0 were classified as “nIBC-like”. Gene Ontology (GO) term enrichment analysis of the resulting DE genes was conducted making use of Database for Annotation, Visualization and Integrated Discovery (DAVID) (http://david.abcc.ncifcrf.gov), while their functional characterization to identify possible enriched molecular networks and canonical pathways was performed using a proprietary software, Ingenuity Pathway Analysis (IPA) from Ingenuity Systems® (http://www.ingenuity.com).

### Construction of transcriptional regulatory network and master regulator analysis

A transcriptional regulation network was inferred using the ARACNe algorithm [15]; it uses an information theoretic approach to dissect physical transcriptional interactions between TFs and their potential targets from mutual information (MI). The MI was estimated by using the “parmigene” package (PARallel Mutual Information calculation for GEne NEtwork reconstruction [20]), available on CRAN. The package provides a parallel estimation of the mutual information based on entropy estimates from *k*-nearest neighbors’ distances with default values of *k* equal to 3, and 10^−12^ for random noise. Master Regulator Analysis (MRA) algorithm [10] was then applied to compute the statistical significance of the overlap between the regulon of each TF (that is, its ARACNe-inferred targets) and the differentially expressed gene list. Given an interaction network, generated by ARACNe, a (candidate) master regulator gene, and a gene signature, the MRA algorithm computes the enrichment of the signature genes in the regulon of that gene*.* The regulon of a TF is defined as its neighborhood in the interaction network. There are two different methods to evaluate the enrichment of the signature in the regulon. One method uses the statistical Fisher’s exact test, while the other approach uses Gene Set Enrichment Analysis (GSEA). In this work, the enrichment was evaluated using the Fisher’s exact test and corrected using the Benjamini and Hochberg (BH) false discovery rate (FDR) for multiple-testing. The significant TFs acting as potential master regulators in IBC and their specific regulons were then imported into the IPA software to identify the most enriched canonical pathways, the over-represented biological processes and molecular functions associated to candidate genes.

### Validation cohort and tissue microarray

A total of 2116 consecutive patients affected by invasive breast carcinoma were collected between January 1992 and December 2006 and included in the database of the Department of Pathology of the G.B. Rossi Hospital in Verona. Clinical data (patient’s medical history, histological diagnosis, staging, treatment) were evaluated as previously published [21]. Briefly, tumor specimens were retrospectively reviewed by pathologists to define the histological size, type and grade of the primary tumor and the histological axillary lymph node status. Clinico-pathological criteria to include a patient in the IBC group (*n* = 39) were carried out according to Manfrin et al. [21]. They included histological diagnosis of neoplastic emboli within superficial dermal lymphatic spaces and/or clinical signs such as diffuse erythema, “peau d’orange”, edema, warmth, tenderness, breast enlargement, and diffuse induration of the breast on palpation, as described by Haagensen [22]. The nIBC group (*n* = 82) consisted of primary invasive breast cancers without any of the above-quoted clinico-pathological criteria of IBC.

Tissue microarrays (TMAs) were constructed from archival tissue blocks of IBC and nIBC samples available in sufficient amount for TMA construction. We used a Beecher tissue microarray instrument (Beecher Instruments, Hacken-sack, NJ, USA). Tissue cylinders, with a diameter of 0.6 mm, were punched from paraffin blocks in demarcated areas on parallel haematoxylin and eosin-stained sections. Three separate cores were sampled from each block, then deposited into a recipient master paraffin block. Each core was placed 1 mm apart on the x-axis and 1.5 mm apart on the *y*-axis of the master block. In total, 6 TMA paraffin blocks were prepared, 3 μm-thick sections were cut from each TMA block and stained with haematoxylin and eosin. Microarray sections were then reviewed to ensure that the sections from each case were morphologically similar to those of the corresponding whole tissue section and represented cancerous or normal epithelial cells. Further 3 μm-thick sections were then cut from each of the master blocks and mounted on super frost plus slides, baked at 60°C for 60 min, deparaffinized, and rehydrated through graded alcohol rinses for immunohistochemical (IHC) analyses.

### Validation cohort and immunohistochemistry

The presence and distribution of tissue polypeptide antigen was visualized by incubation with the specific primary antibody using Leica Bond-Max autostainer system (Milan, Italy). The complete list of primary antibodies and the corresponding experimental conditions are shown in Table 1. All immunohistochemical staining were interpreted regardless of staining intensity by three independent investigators (P.P., A.R. and E.M.) blinded to clinical data and laboratory results. The pattern of immunostaining was recorded according to the number of positive neoplastic cells (at least 1000 total cells were examined) and stratified into two groups: “positive expression”, when more than 5% of tumor cells were positive, and “negative expression”, when less than 5% were positive. The percentage of positive cancer cells, identified by immunoreactivity for each marker, was estimated in triplicate tissue cores. At least three different representative blocks of each case were evaluated to ensure that the staining was homogeneous in the whole tumor. For each tissue section, we also evaluated the distribution of staining, taking into account the positivity in each subcellular compartment as follows: membrane, cytoplasmatic and/or nuclear, respectively. For NFAT5, three distinct patterns of subcellular expression were identified: 1) subcellular cytoplasmic expression (C) was defined as the presence of a homogeneous cytoplasmic staining in all tumour cells; 2) nuclear immunoreactivity (N) corresponded to a homogeneous nuclear staining in tumor cells; 3) cytoplasmic/nuclear immunostaining (N/C) exhibited a mixture of nuclear and cytoplasmic immunohistochemical positivity within tumor cells. For β-catenin, staining was variable both between and within subcellular compartments and classified in 3 groups: 1) (M), in case of complete membranous staining; 2) (M/C), if tumor cells exhibited a mixture of membranous and cytoplasmic positivity; 3) (N/C), if tumor cell nuclei were stained and accompanied by cytoplasmic positivity. Subcellular expression was scored as positive (any positivity) or negative (no staining), by assigning no cutoff value and regardless of intensity. To ensure the reproducibility of the subcellular staining for each marker, one third of the cases were stained a second time. Normal breast tissue cells adjacent to neoplastic cells served as positive internal controls. Tissue specimens from hippocampus and colon carcinoma were used as positive controls for MGA and COX2 immunopositivity, respectively. For MGA, only nucleolar staining was observed, and loss of protein expression was defined as the complete absence of nucleolar expression pattern. E-cadherin (CDH1) staining pattern was evaluated according to Manfrin et al. [21].

**Table 1:** **Primary antibodies used in this study**

**Antigen**	**Incubation time**	**Clone**	**Manufacture**	**Dilution**
NFAT5	15 min	Rabbit polyclonal antibody	ABCAM (AB 110995)	1:100
CDH1	15 min	NCH38 monoclonal antibody	DAKO (M 3612)	1:20
CTNNB1	15 min	15B8 monoclonal antibody	SIGMA (C 7207)	1:150
COX2	15 min	SP21 monoclonal antibody	Thermo LABVISION (RM-9121-S0)	1:50
MGA	60 min	Rabbit polyclonal antibody	ABNOVA (PAB 23917)	1:1000
S1004A	15 min	Rabbit polyclonal antibody	DAKO (A5114)	1:50

### Statistical analysis

Statistical analyses were performed using R and SPSS (version 15.0) for Windows (SPSS Inc., Chicago, Ill.,USA). Data were reported as median or mean and standard deviation (SD), and the mean values compared using the Student’s *t*-test, as indicated. The χ^2^ test was employed to assess the association of gene/protein status and clinico-pathological parameters. For NFAT5 staining pattern, the sensitivity, specificity, positive predictive value (PPV) and negative predictive value (NPV) were computed. Using Kaplan-Meier method we performed survival curves and differences were estimated with the log-rank test. Results were considered statistically significant when a *p* ≤ 0.05 was obtained. Correction of *p*-value was performed with Benjamini and Hochberg false discovery rate method.

### Ethics statement

This study was carried out according to the principles of the Declaration of Helsinki and approved by the Institutional Review Board of Department of Pathology and Diagnosis, University of Verona, Verona, Italy. All patients provided written informed consent for the collection of samples and subsequent analyses.

## Results

### IBC signature is enriched for dis-regulation of cell cycle

In [7], an integrated analysis of gene expression and aCGH microarray applied to IBCs and nIBCs and 13,127 genes identified 24 potential candidate IBC-specific genes that accurately distinguished IBCs and nIBCs. To apply a network-based strategy with a wider gene set and more samples, we derived an IBC signature represented by the genes differentially expressed between 63 IBC samples and 134 nIBC samples. A total of 566 probe-sets were differentially expressed, with a significance threshold of 0.05 and a fold change greater than 1.5, including 206 probe-sets up-regulated in IBC and 360 down-regulated (Additional file 1: Table S1). As expected, the classification of all samples based on the expression of those 566 probe sets (IBC-like or nIBC-like) strongly correlated with the actual IBC-nIBC phenotype (Additional file 2: Figure S1A; *p*-value = 6.1 10^-34^ Student’s *t*-test). We employed DAVID and IPA enrichment analyses of DE probe-sets in an attempt to obtain a biological functional interpretation. Functional enrichment from DAVID analysis showed that the largest fraction of overexpressed genes was enriched for GO terms and KEGG pathway related to the positive regulation of Cell Cycle and in particular the Mitotic Phase. In agreement with these results, Ingenuity Pathway Analysis showed that the most significantly enriched cell functions associated with the overexpressed genes belonged to biological categories such as Cell Cycle, Cellular Assembly and Organization, Cell Death and Survival.

### Validation of the IBC signature

Since all our network-based discovery, reported in the rest of the paper, relies on the above IBC signature, we first validated the signature by using another dataset of 41 IBC and 55 nIBC from the Translational Cancer Research Unit (TRCU) General Hospital Sint-Augustinus Antwerp-Belgium [9]. We computed the centroid expression profile of IBC samples of the learning set and for the 566 selected probesets and evaluate its Pearson correlation, *r,* with each sample of the validation set. As reported in the Additional file 2: Figure S1B, the , the resulting classification (IBC-like or nIBC-like) correlated with the actual IBC-nIBC phenotype (*p*-value of 1.8 10^-4^, Student’s *t*-test). Because of the unbalance between IBCs and nIBCs regarding the SBR tumor grade, we verified that our 566-probe set signature was not more associated with the grade than with the IBC-nIBC phenotype. That was confirmed in multivariate analysis in both the learning and validation sets (Additional file 2: Figure S1 C-D), in which the signature (IBC score in continuous value) and the SBR grade (1, 2, or 3) independently predicted the IBC-nIBC phenotype by using logistic regression.

### Regulatory network derived from gene expression profiles

**Figure 1:**
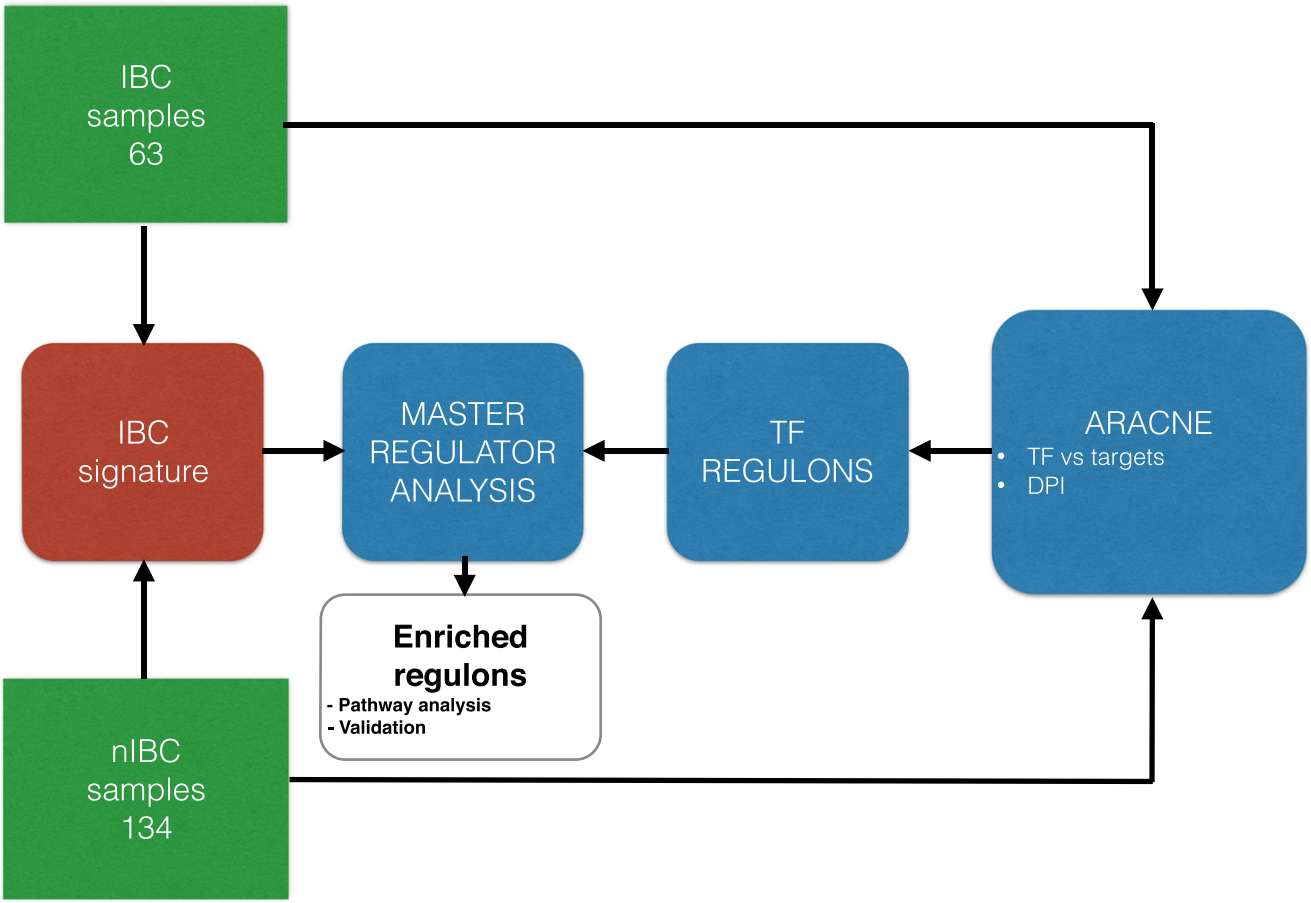
Network inference and MRA flowchart. An overview of the bioinformatics pipeline. Input data are 197 Affymetrix.CEL files (63 IBC samples and 134 nIBC samples). The gene expression datasets were analyzed simultaneously with the ARACNe algorithm to infer a transcriptional regulatory network. Master Regulator Analysis was used to select the TFs showing a significant overlap between the targets in each extracted TF regulon in the network and the IBC gene expression signature. ARACNe comprises two main steps: estimation of mutual information between TFs and potential targets and data processing inequality (DPI) to cut most of the indirect interactions.

To get insight into potential transcriptional regulators of IBC phenotype, we reverse-engineered transcriptional interactions from gene expression data. Reverse-engineering was done using the ARACNe algorithm [15] by applying the analysis pipeline reported in Figure 1. ARACNe is an unbiased algorithm that infers direct transcriptional interactions based on the mutual information between each transcriptional regulator and its potential targets. For optimal analyses, ARACNe requires large data sets of gene expression profiles (at least 100 expression profiles) having significant endogenous (i.e., genetic) and/or exogenous (i.e., perturbation-induced) heterogeneity. We used the whole dataset of expression profiles reported elsewhere by some of the authors [7] which is ideally suited for ARACNe because it is relatively large (n = 197) and diverse. In order to highlight the variability of our dataset, also in terms of clinical features, we report as supplementary figure (Additional file 3: Figure S2) the disease free survival (DFS) curves separated by grade and tumor types showing that they are different given the grade: as expected, the survival of IBC patients was inferior to that of nIBC patients, for both grade II and grade III tumors.

**Figure 2:**
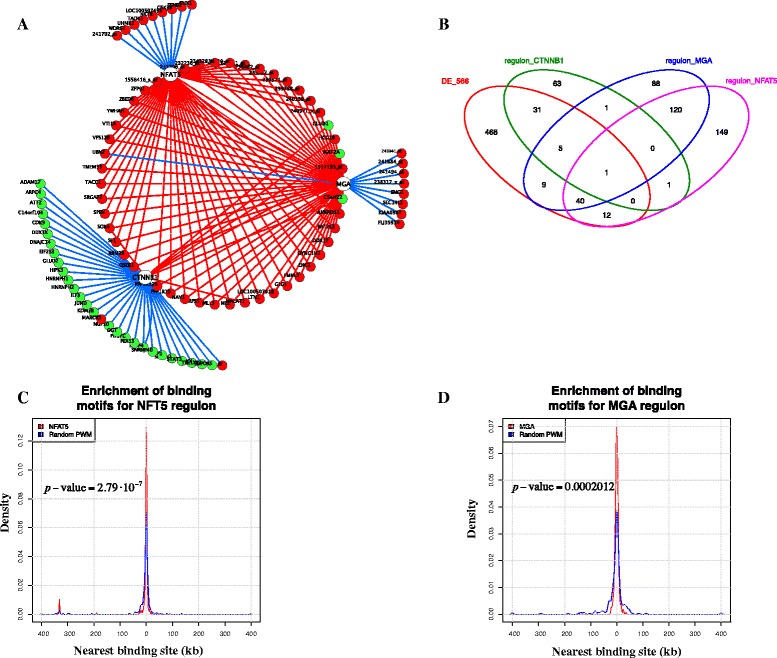
Master Regulators of the IBC signature. **A**: The network shows the top-three MRs (round white nodes) with their respective inferred targets (red or green round nodes). The figure reports just the differentially expressed genes in each regulon. Red nodes depict up-regulated target genes in IBC, and green nodes represent down-regulated targets. The gene network is deeply interconnected, showing a partial overlapping among the three regulons and also gene interactions among the main hubs (edges between white nodes). **B**: Venn diagram of the overlapping between DEGs and the three regulons. **C**: Enrichment of known binding motifs for NFAT5 in its inferred regulon. The occurrence of motif sites is shown as the distance between the TSS of the genes in the regulon and the nearest motif encountered (red line). This was compared with the occurrence of random sites of the same length in the same regulons derived for a random motif. Motifs are taken from Transfac and Human-jolma 2013 databases. **D**: Same as in C for MGA.

ARACNe inferred a network with 81,606 interactions for 1,601 TFs. Master Regulator Analysis [10] was then applied to score the TFs in terms of enrichment with respect to the IBC/nIBC signature. Results of MRA, for each TF, are reported in Additional file 4: Table S2. We chose the top three enriched TFs as genes possibly related to IBC: NFAT5 (Nuclear Factor Of Activated T-Cells 5, *p*-value of MRA = 10^−29^, log fold change of 0.834 and *p*-value = 10^−7^), MGA (MAX Gene Associated, *p*-value of MRA = 10^−35^, log fold change of 0.73 and *p*-value = 10^−10^), and CTNNB1 (Catenin Beta-1, *p*-value of MRA = 10^−33^, log fold change of −1.129 and *p*-value = 10^−15^). NFAT5 and MGA are up-regulated in IBC. Figure 2A reports the interconnected network of these three main hubs, their synergetic effect and a possible mutual “shadow” or an overlap between regulons, suggesting that these MRs might function, at least in part, cooperatively to regulate sets of genes. Figure 2B reports how the DE genes are distributed among the regulons of the selected TFs. Moreover, as was done in [11], we show that the motifs of NFAT5 and MGA are strongly enriched near the promoters of genes in their own regulons (Figure 2C and D). The figures show the distribution of the first occurrence of motifs as functions of the distance from the transcription starting site in the promoter region of each gene in the regulon (red line). Motif occurrences were detected with PWM models of NFAT5 and MGA obtained respectively from Transfac (M00935) and Human-jolma 2013 databases. This was compared with the distribution of the first occurrence of 100 random PWMs of the same length in the same promoter regions (blue line).

One of the most enriched candidates was the Nuclear Factor Of Activated T-Cells 5 (*NFAT5*) gene, a member of the REL family of transcription factors (also known as nuclear factor-κB (NF-κB) family). The NFAT signaling axis is a vertebrate-specific pathway important for various cell functions ranging from the development and activation of lymphocytes to the differentiation of cardiac muscle cells. NFAT5 signaling cascade plays an important role in different diseases and modulating phenotypes associated with malignancy, but less is known about the resulting changes in gene expression that affect breast cancer [23,24]. In our network, NFAT5 showed a regulon of 323 targets (Additional file 5: Table S3) and 53 of them, all up-regulated in IBC, were DE genes. IPA functional and pathway enrichment analysis of NFAT5 regulon revealed 13 significant (corrected *p*-value ≤ 0.05) biological processes, such as Cellular Development (76 genes), Cellular Growth and Proliferation (72), Gene Expression (57 genes), RNA Post-Transcriptinoal Modification (14 genes), Cell Cycle and Morphology (respectively 32 and 42 genes) and four significant (corrected *p*-value ≤ 0.05) enriched pathways belonging to categories of Actin Cytoskeleton Signaling (10 genes), Insulin Receptor Signaling (7 genes), RhoGDI Signaling (8 genes) and Protein Kinase A Signaling (12 genes).

In a number of systems, a crosstalk between NFAT5 and WNT/β-catenin signaling through its interaction with β-catenin transactivation C-terminal domain has been reported [25]. Accordingly, our analysis showed a significant enrichment in genes of the CTNNB1 gene expression network. This gene codes for β-catenin, a key downstream mediator of WNT canonical signaling pathway [26]. Aberrant activation of the WNT/β-catenin cascade is linked to a wide range of biological processes leading to stem cell expansion and disturbed tissue architecture [27,28]. In our network, the CTNNB1 regulon counted 102 members, and 37 of them were enriched in the differentially expressed gene list (corrected *p*-value < 10^−33^). Thirty-one of these 37 genes were down-regulated in IBC. The IPA functional enrichment analysis of the CTNNB1 regulon pinpointed the metabolic pathway associated with D-glutamine and D-glutamate, which is supported by a significant (corrected *p*-value ≤ 0.05) enrichment of biological functions related to regulation of Gene Expression (30 genes), Cellular Growth and Proliferation (41 genes) and Cellular Movement (15 genes).

Our model system showed that our gene signature was also centered on the MGA gene that codes for a transcription factor of the T-box/MYC families. MGA contains both a T-box and a basic helix-loop-helix leucine zipper (bHLH-zip) domain and is part of the network of MAX and MAX-interacting proteins, which are involved in fundamental aspects of cell-fate decisions [29]. The biological roles of MGA in some types of cancer such as lymphocytic leukemia and lung cancer are beginning to emerge through extensive molecular profiling studies [30,31]. In our regulatory network reconstruction, MGA was connected to a regulon of 264 target genes, and 55 of them were enriched in the differentially expressed gene list (corrected *p*-value < 10^−35^). Fifty-two of these 55 genes were up-regulated in IBC. IPA analysis indicated that the members of the MGA regulon are associated with 15 significant (corrected *p*-value ≤ 0.05) functional categories including Gene Expression (49 genes), Cellular Development (46 genes), Cellular Growth and Proliferation (53 genes), DNA Replication, Recombination and Repair (11 genes), and Cellular Assembly and Organization (20 genes) among over-represented functions. By zooming on the specific molecular pathways associated with MGA target genes, data mining through IPA showed 44 canonical pathways. Notably, Insulin Receptor Signaling (6 genes), Actin Cytoskeleton Signaling (7 genes), ERK5 Signaling (4 genes), Phospholipase C Signaling (7 genes) and Estrogen Receptor Signaling (5 genes) were the five most enriched pathways (Fisher’s exact test *p*-value ≤ 0.05), while the top scoring disease-related pathway was that one relative to Cancer (145 genes).

### NFAT5 is a novel marker of IBC

To validate the top enriched MRs on independent samples, we used TMAs comprising 39 IBCs, 82 nIBCs and 15 benign breast tissues. We first verified that the two groups of IBC and nIBC differed in terms of disease-specific survival. By using Kaplan-Meier method, we confirmed that IBC patients had a poorer prognosis than nIBC patients accounting for a median of survival of 60.4 and 147.4 months, respectively (*p* < 0.0001) (Additional file 6: Figure S3A). A detailed description of clinicopathological characteristics of IBC and nIBC samples has already been provided in our previous report [21]*.* Globally, the clinical and pathological findings were coherent with the literature.

**Figure 3:**
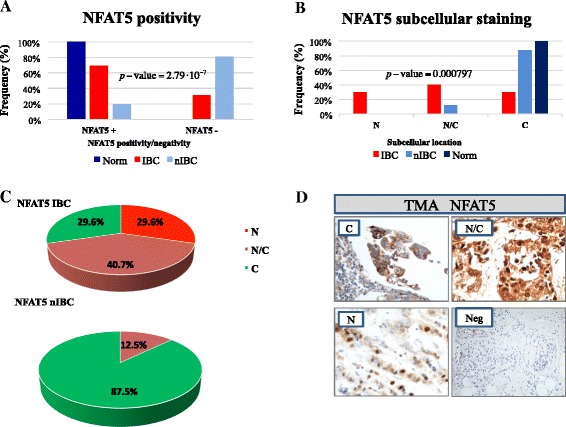
Expression of NFAT5 protein in normal and cancer breast specimens spotted on TMA. NFAT5 protein staining was evaluated by immunohistochemistry in 39 IBC, 82 nIBC and 15 normal breast specimens (Norm). **A**: The percentage of NFAT5-positive and NFAT5-negative cases. **B**-**C**: Percentage of NFAT5 subcellular staining detected on TMA validation series. The *p*-values reported in each graph were obtained by chi-square test with Yates’s correction for continuity between IBC and nIBC group. **D**: NFAT5 subcellular immunostaining pattern: cytoplasmic (C), nuclear/cytoplasmic (N/C), nuclear (N) or negative (Neg).

We measured the protein expression of our IBC candidate genes and their distribution in subcellular compartments (nuclear, cytoplasmic/nuclear, membrane, or membrane/cytoplasmic) in this sample series. We observed that NFAT5 immunostaining was always positive and localized in the cytoplasm in all benign mammary epithelial tissues (15 out of 15; 100%; Figure 3A-B and Additional file 6: Figure S3B). The percentage of samples NFAT5-positive was higher in IBCs (27 out of 39; 69.2%) than in nIBCs (16 out 82; 19.5%; Figure 3A). Therefore, if we consider NFAT5-positivity as a marker of IBC we get 62.8% positive predictive value (PPV) and 84.6% of negative predictive value (NPV). Next, we classified the NFAT5 subcellular staining pattern into three categories: cytoplasmic, nuclear/cytoplasmic and nuclear (Figure 3D). By applying this criterion, we observed that nuclear or nuclear/cytoplasmic positivity was higher in IBC than in nIBC samples (70% vs. 12.5%, *p*-value = 0.000797; Figure 3C). These data indicated that most NFAT5-positive IBCs (19 out of 27) were characterized by a marked nuclear staining often associated with cytoplasmic immunoreactivity. Notably, in IBC, NFAT5 nuclear staining showed a high sensitivity and specificity of 70% and 88% respectively. By contrast, only 2 out of 16 (12.5%) of NFAT5 positive nIBC specimens showed nuclear immunoreactivity, while the majority (14 out of 16; 87.5%) showed cytoplasm positivity (Figure 3B-C). Summarizing, the nuclear expression of NFAT5 has a 90.5% of PPV and 63.6% of NPV. As shown above at the mRNA level, the NFAT5 protein positivity was significantly different between IBC and nIBC independently of the tumor grade: in the grade 1–2 samples (14 IBCs and 56 nIBCs), NFAT5 staining was positive in 9 IBCs (64%) and in 10 nIBCs (18%; *p*-value = 4.7 10^-4^, χ^2^ test), and in the grade 3 samples (22 IBCs and 14 nIBCs), NFAT5 staining was positive in 16 IBCs (73%) and in 2 nIBCs (14%; *p*-value = 6.3 10^-4^, χ^2^ test). These data suggested that a nuclear accumulation of NFAT5 plays a relevant role in IBC pathogenesis.

### Crosstalk between NFAT5 and WNT/β-catenin signaling

**Figure 4:**
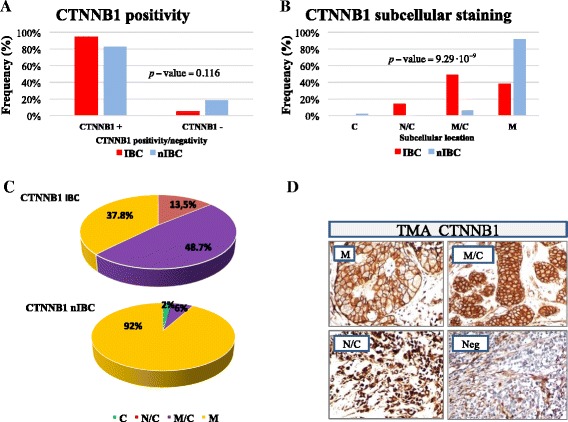
Expression of CTNNB1 protein in IBC and nIBC specimens. **A**: The percentage of CTNNB1-positive and CTNNB1-negative cases in the validation series comprising 39 IBC and 80 nIBC specimens. **B**-**C**: Percentage quantification of CTNNB1 staining pattern detected on TMA validation series. The *p*-values reported in each graph were obtained by chi-square test with Yates’s correction for continuity between IBC and nIBC groups. **D**: CTNNB1 subcellular immunostaining pattern: membrane (M), membrane/cytoplasmic (M/C), nuclear/cytoplasmic (N/C) or negative (Neg).

In IBC E-cadherin expression is maintained in the primary tumor and tumor emboli [6-8]. Accordingly, in the validation datasets, we found that E-cadherin positivity was observed in almost all cases of IBCs (data not shown), its expression, however, was not statistically different in comparison to nIBCs (87.2% vs. 82.5%; *p*-value = 0.698). Moreover, β-catenin positivity was prevalent in IBCs but not statistically significant when compared with nIBCs (95% vs. 82.5%; *p*-value = 0.116; Figure 4A). IHC staining of normal breast specimens, showed that β-catenin was expressed in mammary epithelial cells and mainly restricted to the plasma membrane as well as E-cadherin (data not shown). To further characterize the relationship between WNT canonical pathway and IBC, we analyzed β-catenin expression pattern by taking into account membrane, membrane/cytoplasmic, and nuclear/cytoplasmic localization (Figure 4D). IBCs frequently showed nuclear/cytoplasmic and/or membrane/cytoplasmic positivity for β-catenin as compared to nIBCs (62.1% vs. 7.6%; *p-*value *=* 9.29 10^-09^; Figure 4B-C), revealing a significant association between β-catenin aberrant expression and IBC phenotype. In addition, a positive correlation between E-cadherin and β-catenin membrane expression was found in nIBCs, suggesting that a preserved E-cadherin/β-catenin complex on the membrane is a pattern frequently observed in nIBCs (Additional file 6: Figure S3 C-D). Collectively, these data provide suggest that WNT/β-catenin canonical activation is preferentially found in IBC.

**Figure 5:**
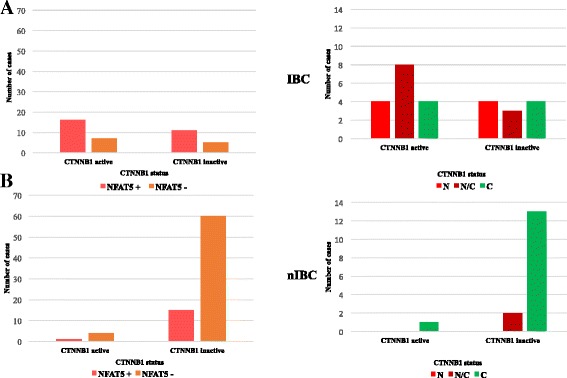
Crosstalk between NFAT5 and WNT/CTNNB1-signaling in IBC pathogenesis. **A**-**B**: NFAT5 positivity and its subcellular distribution according to the CTNNB1 activation in IBC and nIBC TMA validation series, respectively. CTNNB1 inactive indicates negative and/or membrane staining; CTNNB1 active indicates cytoplasmic, nuclear and/or nuclear/cytoplasmic accumulation. Abbreviations: cytoplasmic (C), nuclear (N) and nuclear/cytoplasmic (N/C).

In addition, we wanted to determine the possible relationships between WNT/β-catenin and NFAT5 pathway. In the IBC subgroup, NFAT5 positivity and its nuclear and/or nuclear/cytoplasmatic accumulation was independent from the inactive (negative and/or membrane) or active (cytoplasmic and/or nuclear) β-catenin state (Figure 5A). In the nIBC subgroup, negative NFAT5 expression was closely correlated to inactive form of β-catenin but not with an activated status of WNT/β-catenin signaling (Figure 5B). These findings suggested that activation of NFAT5 signaling operate, at least partially, regardless of WNT/ β-catenin activation pathway, to promote IBC development and progression.

### Validation of NFAT5 target genes

**Figure 6:**
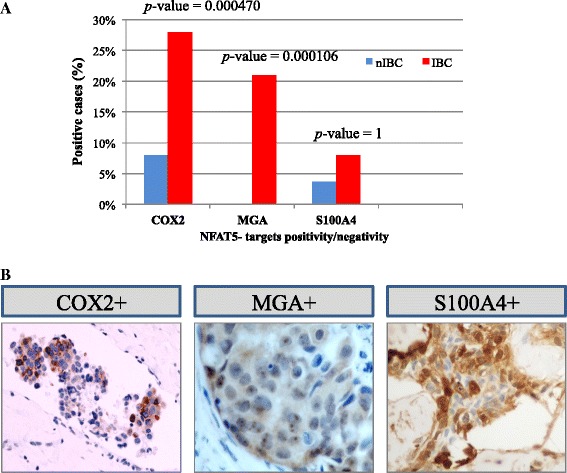
MGA and NFAT5-target genes expression in the TMA validation series. **A**: Percentage of tumor specimens expressing MGA and NFAT5-target genes (COX2 and S100A4) in the TMA validation series of IBC and nIBC subtypes. The *p*-values reported in graph were obtained by chi-square test with Yates’s correction for continuity between IBC and nIBC groups. **B**: COX2, MGA and S100A4 immunopositivity in representative cases of IBC.

Since *in-silico* results also suggested a putative involvement of MGA and other NFAT5-target genes in IBC pathogenesis, we wondered whether NFAT5-target genes may be correlated with that of the top deregulated genes in our validation cohort. To this end, we selected two additional genes (COX2 and S100A4), experimentally validated NFAT5- target genes in breast and other cellular models [32,33]. Interestingly, COX2 positivity and MGA positivity were significantly more prevalent in IBCs than in nIBCs supporting *in-silico* and literature data. In contrast, S100A4 did not show any significant difference between IBC and nIBC (Figure 6A and B). These results confirmed, at least partially, that the top deregulated gene MGA and COX2 genes were related to NFAT5 expression pattern in IBC subgroup.

## Discussion

Although IBC is the most aggressive form of breast cancer, very few studies have identified IBC-specific gene signatures or clinically applicable biomarkers, and therapeutic approaches are still based on clinic-pathological factors similar to those used for nIBC patients. In this study, we employed a systems biology approach with a network-based strategy to gain insight into pathways and master regulators associated with IBC pathogenesis. By applying this approach to two gene expression datasets followed by validation at the protein level, we identified NFAT5 as a novel biomarker that potentially might be developed into clinical assays to improve IBC classification into molecularly distinct phenotype.

The roles of NFAT transcription factors have been extensively studied in the immune system but their impact in human cancer remains poorly understood. Ubiquitous expression of NFAT isoforms in mammalian tissues has been described, and mainly two isoforms, NFAT1 and NFAT5, have been reported as overexpressed in human invasive ductal breast carcinomas. Overall, the contribution of specific NFAT isoforms in distinct BC phenotypes is still unknown. We show that the constitutive activation of NFAT5 signaling in IBC might, at least in part, explain the aggressiveness of the IBC phenotype. The transcriptional activity of NFATs is primarily regulated by post-translational modifications that in turn determine the subcellular localization. In the basal state, two kinases - tyrosine phosphorylation-regulated kinase 2 (DYRK2) and casein kinase 1 (CK1) - phosphorylate NFAT TFs, maintaining them localized to the cytoplasm in an inactive conformation: the nuclear translocation and transcriptional activation of NFAT in cancer cells lead to the induction of genes that promote tumor progression, migration and invasion [24]. In line with this, our analysis revealed that NFAT5 is expressed in non-neoplastic breast tissues and exclusively confined to the cytoplasmic compartment. Interestingly, in our validation series of malignant tissues, the pattern of NFAT5 expression was markedly different between IBC and nIBC phenotypes. In fact, we observed an overexpression of NFAT5 in IBCs as compared to nIBCs, which was further supported by a significantly higher nuclear distribution in IBC than nIBC. These results suggest that increased NFAT5 transcriptional activity or “constitutive activation” might play a causal role in IBC pathogenesis. Although the molecular mechanisms underlying the nuclear translocation of NFAT5 were not explored in the present study, our data show that IBC and nIBC phenotypes are biologically distinct and NFAT5 could serve as surrogate biomarker in an immunohistochemical assay.

The algorithm also identified CTNNB1 as one of the top three enriched TFs possibly related to IBC. Aberrant WNT/β-catenin signaling has been reported in a variety of tumors including breast carcinomas [34], but its prevalence remains debated. Nuclear β-catenin expression has been found in triple-negative/basal-like breast carcinomas and associated with poor clinical outcome, while there is currently no information available on β-catenin expression in IBC [27,28]. By using our validation series, we showed that nuclear or cytoplasmic expression of β-catenin is more recurrent in IBCs than in nIBCs. Even more interestingly, we found evidence that altered expression or activation of NFAT5 occurs independently of the nuclear β-catenin accumulation, suggesting that a substantial portion of biological responses in IBC may be mediated by NFAT5 transcriptional network. In addition, negative and or cytoplasmic NFAT5 expression was accompanied by “normal membranous” β-catenin localization in nIBCs, supporting the hypothesis that the better prognosis of nIBC subtype is associated with a concomitant inactivation of signaling. The crosstalk between WNT pathway and NFAT transcriptional activity, however, warrants further studies in *in vitro* and *in vivo* systems.

To support the importance of NFAT5 transcriptional activation in IBC and further prove its biological relevance in IBC, we also studied in our TMAs validation series MGA and two known NFAT5 target genes, COX2 and S100A4. Notably, MGA and COX2 were almost exclusively expressed in a proportion of IBC, reinforcing the role of NFAT5 signaling as a central player of IBC progression and providing further support to our *in-silico* findings.

## Conclusion

We applied a network-based strategy to uncover novel MRs underlying IBC pathogenesis. We discovered that NFAT5 transcription factor could constitute a surrogate marker of NFAT-signaling pathway activation of IBC and potentially a guide to assignment of IBC-specific therapeutic agents. Our results indicate that NFAT5 pathway activation might be a potential and specific player of IBC progression. Since antagonists of the NFAT transcription factors family have anti-tumor-promoting activity, our results may be relevant to the assessment of new investigational drugs in preclinical trials and in turn guide “personalized” therapeutic trial dedicated to IBC.

## Additional files

Additional file 1: Table S1. List of 566 probe sets differentially expressed between IBC samples and nIBC samples. Additional file 2: Figure S1. Validation of the IBC signature and independence from the SBR grade. A-B: The IBC score is a predictor of the IBC phenotype in the IPC cohort (learning set: A) and the TCRU cohort (validation set: B). C-D: Univariate and multivariate analyses of IBC score (continuous value) and SBR grade (1, 2, or 3) for predicting the IBC-nIBC phenotype in the IPC series (C) and in the TCRU series (D). Additional file 3: Figure S2. Survival curves of the IBC and nIBC patients in the GSE23720 dataset according to tumor grade (II and III). Kaplan-Meier curves are shown and compared with the log-rank test. Additional file 4: Table S2. List of MRs tested and ranking in the Master Regulator Analysis. Additional file 5: Table S3. List of genes associated with the NFAT5, MGA and CTNNB1 regulons. Additional file 6: Figure S3. Survival analysis in TMA validation series, NFAT5 staining pattern and correlation between E-cadherin and β- staining patterns. A: Kaplan-Meier analysis of IBC and nIBC patients in TMA validation series. The curves indicates that IBC group differs significantly from nIBC group with respect to the diseases specific survival. B: Immunostaining pattern of NFAT5 at high magnification (60x) in representative cases of normal breast and IBC core specimens. NFAT5 staining is distributed in the cytoplasmic compartment (brown) of normal mammary epithelial cells but does not mark the nuclei (blue) in the normal mammary epithelial cells. In IBC, NFAT5 marks both nuclei and cytoplasm (brown). C, D: Subcellular distribution of β-catenin in relation to E-cadherin expression in nIBC and IBC TMA validation series, respectively. β-catenin inactive indicates negative and/or membrane staining; β-catenin active indicates cytosolic and/or nuclear accumulation.

## Abbreviations

IBC: Inflammatory breast cancer
nIBC: Non-inflammatory breast cancer
BC: Breast cancer
ER: Estrogen receptor
PR: Progesterone receptor
TNBC: Triple-negative breast cancer
TP53: Tumor protein p53
CDH1: Cadherin 1 or E-cadherin
VEGF: Vascular endothelial growth factor
FGF2: Fibroblast growth factor 2
VEGFR1: Soluble vascular endothelial growth factor receptor-1
Rho C GTPase: Ras homolog family member C
WISP3: WNT1 inducible signaling pathway protein 3
LIBC: Lost in Inflammatory breast cancer
Ki-67: Marker of proliferation Ki-67
aCGH: Array-comparative genomic hybridization
TGFβ: Transforming growth factor beta 1
TF: Transcription factor
MR: Master regulator
STAT3: Signal transducer and activator of transcription 3
CEBP/B: CCAAT/enhancer binding protein/beta
NFAT5: Nuclear factor of activated T-cells 5
GEO: Gene expression omnibus
RMA: Robust multi-array average
DE: Differentially expressed
GO: Gene ontology
DAVID: Database for annotation, visualization and integrated discovery
IPA: Ingenuity pathway analysis
ARACNe: Algorithm for the reconstruction of accurate cellular networks
MI: Mutual information
parmigene: Parallel mutual information estimation for gene network reconstruction
MRA: Master regulator analysis
GSEA: Gene set enrichment analysis
BH: Benjamini & Hochberg
FDR: False discovery rate
TMA: Tissue microarray
IHC: Immunohistochemistry
MGA: MAX gene associated
COX2: Cyclooxygenase-2
SPSS: Statistical package for social science
SD: Standard deviation
PPV: Positive predictive value
NPV: Negative predictive value
KEGG: Kyoto encyclopedia of genes and genomes
DFS: Disease free survival
CTNNB1: Catenin beta 1 or β-catenin
TSS: Transcription starting site
bHLH-zip: Basic helix-loop-helix leucine zipper
MAX: MYC associated factor X
S100A4: S100 calcium-binding protein A4 or metastasin
NFAT1 (or NFATC2): Nuclear factor of activated T-cells
cytoplasmic: Calcineurin-dependent 2
DYRK2: Dual specificity tyrosine-phosphorylation-regulated kinase 2
CK1: Casein kinase 1
