# Supplementary material for: Systems biology analysis reveals NFAT5 as a novel biomarker and master regulator of inflammatory breast cancer
Source: J Transl Med. 2015 May 1;13:138. doi: 10.1186/s12967-015-0492-2 (PMC4438533; doi:10.1186/s12967-015-0492-2)
Supplement: Additional file 2: Figure S1. — Validation of the IBC signature and independence from the SBR grade. A-B: The IBC score is a predictor of the IBC phenotype in the IPC cohort (learning set: A) and the TCRU cohort (validation set: B). C-D: Univariate and multivariate analyses of IBC score (continuous value) and SBR grade (1, 2, or 3) for predicting the IBC-nIBC phenotype in the IPC series (C) and in the TCRU series (D). [file 12967_2015_492_MOESM2_ESM.pdf]

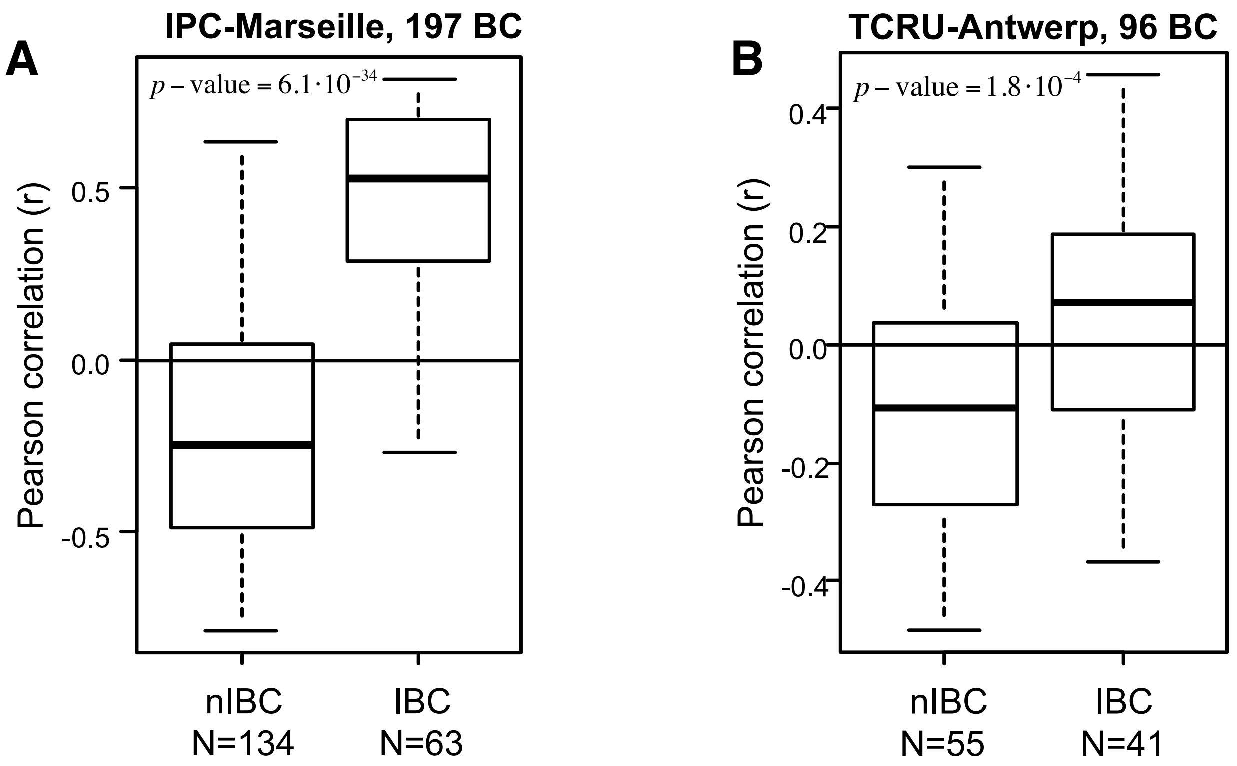

C

IPC-Marseille, 197 BC

	Univariate			Multivariate		
	N	Odds Ratio [C.I.95%]	p-value	N	Odds Ratio [C.I.95%]	p-value
IBC score, r	197	374 [106-1.6e3]	$1.27 * 10^{-12}$	191	309 [76.3-1.7e3]	$7.07 * 10^{-10}$
SBR grade	191	9.21 [5.17-17.7]	$2.68 * 10^{-09}$	191	7.87 [3.58-19.43]	$5.47 * 10^{-05}$

D

TCRU-Antwerp, 96 BC

	Univariate			Multivariate		
	N	Odds Ratio [C.I.95%]	*p*-value	N	Odds Ratio [C.I.95%]	*p*-value
IBC score, r	96	41.5 [7.57-271]	$5.69 * 10^{-04}$	94	24.0 [3.75-179]	$6.49 * 10^{-03}$
SBR grade	94	3.75 [2.17-6.94]	$1.74 * 10^{-04}$	94	3.14 [1.76-5.94]	$1.90 * 10^{-03}$
