# Supplementary figures and images for: Systems biology analysis reveals NFAT5 as a novel biomarker and master regulator of inflammatory breast cancer
Source: J Transl Med. 2015 May 1;13:138. doi: 10.1186/s12967-015-0492-2 (PMC4438533; doi:10.1186/s12967-015-0492-2)

## Kaplan-Meier Plots

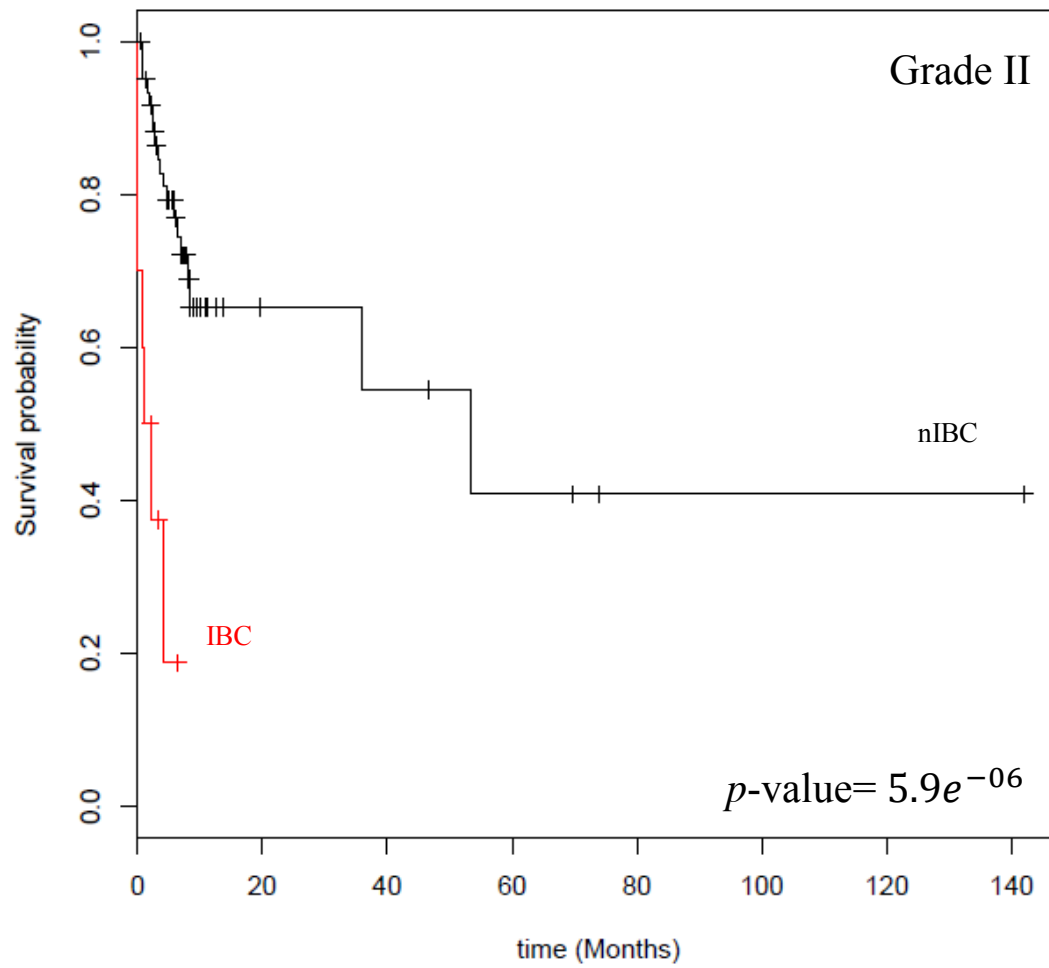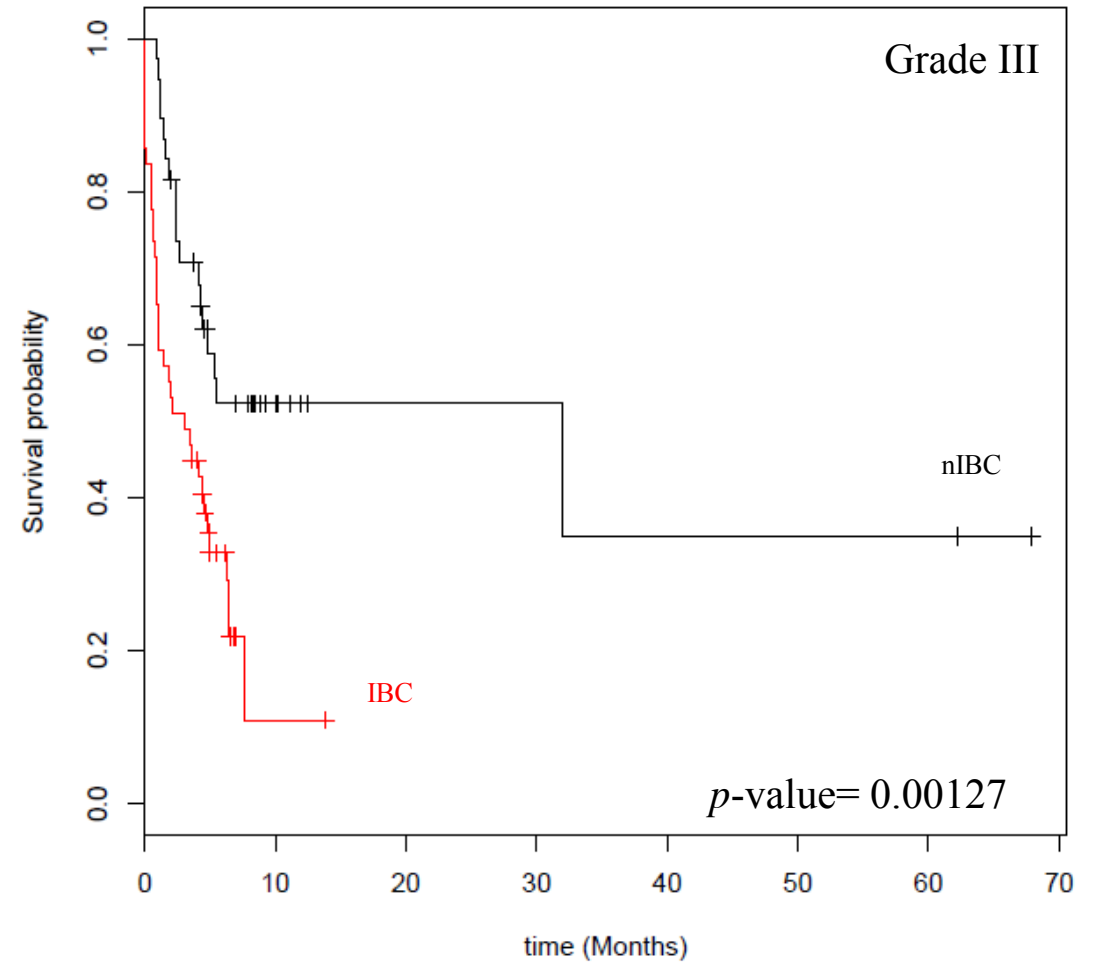

Supplement: Additional file 3: Figure S2. — Survival curves of the IBC and nIBC patients in the GSE23720 dataset according to tumor grade (II and III). Kaplan-Meier curves are shown and compared with the log-rank test. [file 12967_2015_492_MOESM3_ESM.pdf]

**A**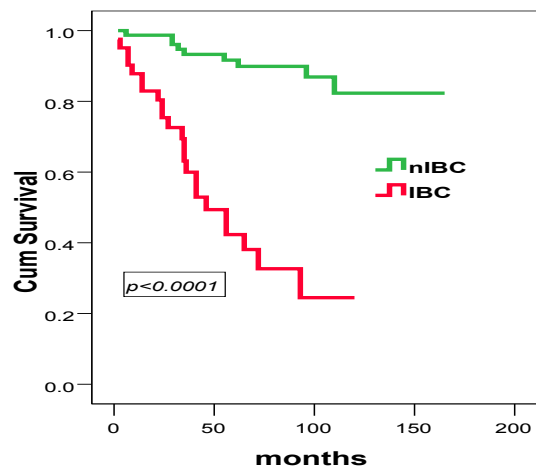**B**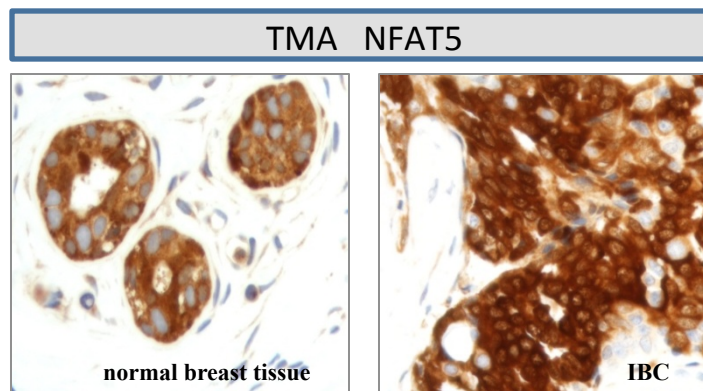**C****nIBC**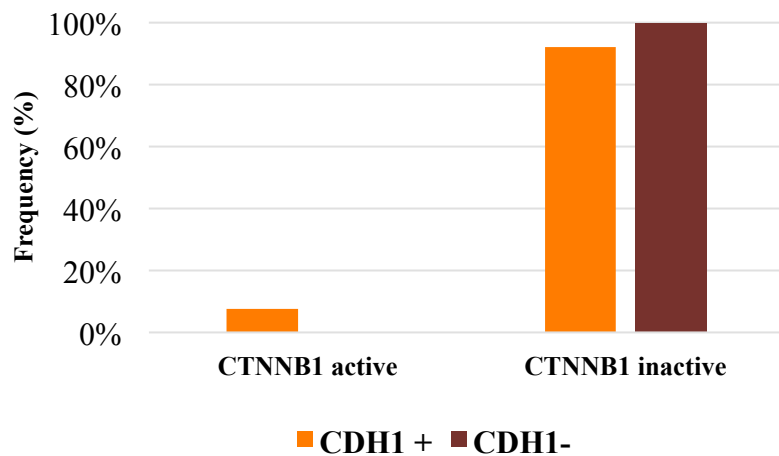**D****IBC**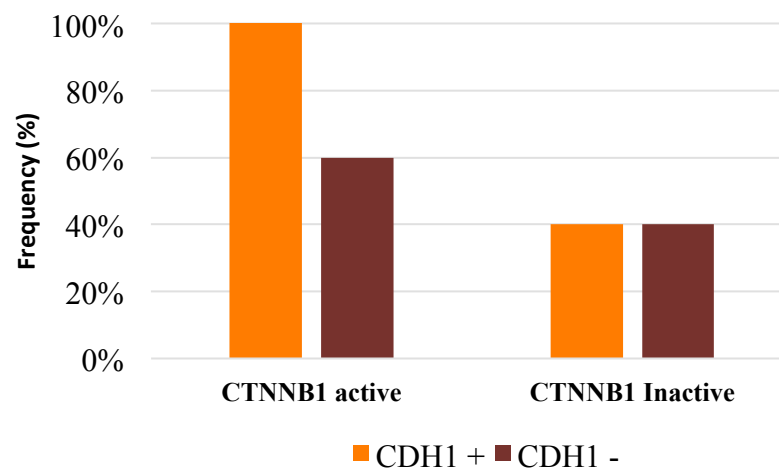

Supplement: Additional file 6: Figure S3. — Survival analysis in TMA validation series, NFAT5 staining pattern and correlation between E-cadherin and β- staining patterns. A: Kaplan-Meier analysis of IBC and nIBC patients in TMA validation series. The curves indicates that IBC group differs significantly from nIBC group with respect to the diseases specific survival. B: Immunostaining pattern of NFAT5 at high magnification (60x) in representative cases of normal breast and IBC core specimens. NFAT5 staining is distributed in the cytoplasmic compartment (brown) of normal mammary epithelial cells but does not mark the nuclei (blue) in the normal mammary epithelial cells. In IBC, NFAT5 marks both nuclei and cytoplasm (brown). C, D: Subcellular distribution of β-catenin in relation to E-cadherin expression in nIBC and IBC TMA validation series, respectively. β-catenin inactive indicates negative and/or membrane staining; β-catenin active indicates cytosolic and/or nuclear accumulation. [file 12967_2015_492_MOESM6_ESM.pdf]
